# Enhancing All‐Solid‐State Batteries Performance Through Thickness Control and Surface Passivation of Thermally Evaporated Lithium Metal Anodes

**DOI:** 10.1002/advs.76456

**Published:** 2026-07-13

**Authors:** Jinsong Zhang, Linfeng Xu, Robin N. Wullich, Thomas J. Schmidt, Mario El Kazzi

**Affiliations:** ^1^ PSI Center for Energy and Environmental Sciences Paul Scherrer Institute Villigen Switzerland; ^2^ Institute For Molecular Physical Science ETH Zurich Zurich Switzerland

**Keywords:** all‐solid‐state batteries, cycling performance, lithium metal anodes, passivation layer, thermal evaporation

## Abstract

Thin lithium metal anodes are key to realizing high‐energy‐density and enhanced safety in all‐solid‐state batteries (ASSBs). However, extruded lithium foils below 50 µm suffer from poor structural integrity, rough surfaces, and resistive, native passivation layers, which limit cycling stability. In this study, thermally evaporated, high‐purity lithium with smooth grain‐boundary surface morphology significantly improves interfacial contact and electrochemical performance. Replacing extruded lithium with a 50 µm evaporated lithium anode increases the critical current density (CCD) from 1.6 to 2.1 mA cm^−2^ and enables 161 cycles at 1.5 mA cm^−2^ and 1.6 mAh cm^−2^. A thickness‐dependence study reveals that maintaining a sufficient lithium reservoir is crucial to mitigate void formation and compensate for solid electrolyte interphase (SEI) growth. To overcome limitations at reduced thickness, an ultra‐thin 65 nm LiF passivation layer was applied to 25 µm evaporated lithium. The LiF coating suppresses chemical degradation during storage, stabilizes lithium/LPSCl interface, limits SEI growth, and mitigates dendrite, increasing the CCD to 2.6 mA cm^−2^. In full cells, LiF‐coated 25 µm lithium delivers over 500 and 300 cycles at current densities of 1.5 and 3 mA cm^−2^ respectively. These results establish LiF‐passivated thermally evaporated lithium as a high‐performance anode design for next‐generation ASSBs.

## Graphical abstract

This study demonstrates that thermally evaporated lithium anodes combined with LiF passivation provide a high‐performance thin anode design for next‐generation ASSBs. This approach increases the critical current density from 1.6 to 2.6 mA cm^−2^, enabling full cells to achieve over 500 and 300 cycles at 1.5 and 3 mA cm^−2^, respectively, outperforming even twice‐as‐thick commercially extruded lithium (50 µm).

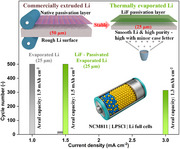

## 1 Introduction

Thin metallic lithium, with its exceptionally high theoretical capacity (3860 mAh g^−1^) and low redox potential of −3.04 V (vs. standard hydrogen electrode), remains one of the most promising anodes materials for achieving high energy density in all‐solid‐state batteries (ASSBs) [1, 2]. Despite these advantages, the practical implementation of lithium metal anodes faces significant challenges. During cycling, inhomogeneous lithium plating and stripping, together with interfacial void formation, can initiate dendrite growth. These dendrites may penetrate through pore‐percolation pathways and generate plating‐induced mechanical cracks within the solid electrolyte (SE), ultimately causing internal short circuits and cell failure [3, 4, 5]. In addition to these morphological instabilities, the high chemical reactivity of lithium combined with the narrow electrochemical stability window of sulfide‐based SE such as Li_6_PS_5_Cl (LPSCl), results in severe interfacial side reactions consuming active lithium and decomposing LPSCl, which increases interfacial resistance, degrades performance, and accelerates capacity fade [6, 7, 8].

Therefore, maintaining the structural integrity of the plated/stripped lithium, while simultaneously stabilizing the SE/lithium metal interface, is crucial for achieving long‐term, high‐performance cycling of lithium metal ASSBs under demanding operating conditions [9, 10].

Commercial lithium metal foils are currently produced through extrusion and calendaring techniques, enabling reliable large‐scale production of films with thicknesses down to approximately 50 µm [1]. However, further thickness reduction is constrained by the intrinsic softness and the strong adhesiveness of lithium. These properties complicate mechanical handling, promote undesired deformation during processing, and ultimately increase manufacturing cost for ultra‐thin lithium foils [11]. In addition, extruded lithium rapidly develops a native surface layer rich in Li_2_CO_3_, Li_2_O, and LiOH; even under dry‐room condition. This layer is mechanically fragile, and electrically insulating, leading to poor interfacial contact and increased resistance. The use of roll‐pressing lubricants during calendaring introduces additional surface contaminants, which further degrade interfacial stability and negatively affect overall battery performance. Alternative, liquid‐based processing approaches are currently under development and face similar limitations. Achieving uniform lithium metal below 50 µm remains challenging due to the intrinsically low wettability of molten lithium on most substrates, which results in poor film uniformity at low thicknesses. Moreover, these processes must be carried out under strict inert‐atmosphere conditions due to the high lithium chemical reactivity [12]. Electrodeposition represents another potential route; however, it typically yields highly porous and mossy morphology that is unsuitable for battery applications [13].

In contrast, thermal evaporation of high‐purity lithium under vacuum has emerged as a promising, controllable and cost‐effective approach for producing ultra‐thin lithium films. This method enables the deposition of homogeneous, smooth and compact lithium films with thicknesses below 10 µm. Such vapor‐deposited films eliminate many of the interfacial and morphological issues associated with mechanically processed or liquid‐processed lithium. As reported by Pasta et al. [14]. the techno‐economic analysis further underscores the importance of thin lithium layers, identifying 17 µm as the critical lithium thickness required to achieve high volumetric energy density of battery (1000 Wh l^−1^) at a manufacturing cost of US$24.2 kWh^−1^. This underscores the urgent need for scalable, reliable, and contamination‐free fabrication techniques for ultra‐thin lithium metal films.

Previous studies have highlighted the advantages of thermally evaporated lithium as an anode material. Ingenito et al. reported lower charge‐transfer resistance and more uniform lithium via thermal evaporation compared with extruded lithium, resulting in enhanced cycling performance [15]. Cras et al. further revealed that lithium films thinner than 20 µm undergo rapid lithium depletion due to extensive solid electrolyte interphase (SEI) formation and the accumulation of electrochemically inactive “dead” lithium. These finding emphasize the need to engineer ultra‐thin (< 20 µm) lithium layers with appropriate protective coating to mitigate interfacial degradation [16].

Despite the widespread use of evaporated lithium in research and prototyping because of its high purity, smooth morphology and controllable thickness, a comprehensive understanding of its chemical evolution, mechanical stability, and interfacial resistance in ASSBs configurations remains absent. Previous studies primarily focused on fabrication strategies and electrochemical performance in liquid‐electrolyte systems [14, 15, 16], where evaporated lithium was shown to improve interfacial uniformity compared with mechanically processed lithium. However, these conclusions cannot be directly transferred to ASSBs, in which interfacial chemistry, mechanical constraints, and ion transport behavior differ substantially. In particular, the surface aging behavior of evaporated lithium has received limited attention, despite the high reactivity of freshly deposited lithium and its tendency to gradually form insulating surface species.

In ASSBs, additional critical challenges persist, including interfacial contact loss during cycling at high current densities and areal capacities [17], as well as the formation of non‐beneficial interphases containing Li_2_S, LiCl, and Li_3_P. These interphases impede Li‐ion transport, increase interfacial resistance, and accelerate performance degradation [18]. Moreover, despite the improved morphology of evaporated Li, ultrathin Li electrodes still suffer from void formation, contact loss, and dendrite growth during cycling. The effects of lithium thickness and the effectiveness of passivation or buffer layers have also not been systematically evaluated. Addressing these knowledge gaps is essential for enabling the reliable operation of thermally evaporated ultra‐thin lithium anodes in practical ASSB architectures.

In this study, we investigated and compared three types of lithium metal anodes: commercially extruded lithium, thermally evaporated lithium by physical vapor deposition (PVD) technique, and LiF‐passivated evaporated lithium. First, we examine the evolution of surface morphology and chemical composition over storage time to understand the intrinsic chemical stability and reactivity of each lithium source. Second, we evaluate the electrochemical performance of evaporated lithium films with varying thickness ranging from 12.5 to 50 µm in both Li|Li symmetric cells and LiNi_0.8_Co_0.1_Mn_0.1_O_2_ (NCM811)|Li full cells configurations using Li_6_PS_5_Cl (LPSCl) as solid electrolyte. These experiments allow us to establish the correlation between lithium film thickness, voids and inactive lithium formation. Third, we introduce and optimize thin LiF passivation layers to mitigate the Li/SE interfacial reactions and suppress dendrites growth. Overall, our results show that the optimized 25 µm evaporated lithium film, when combined with 65 nm LiF passivation, delivers markedly improved interfacial stability and cycling performance compared to the commercially extruded lithium metal.

## 2 Results and Discussion

### 2.1 Comparison of PVD‐Deposited and Commercially Extruded Lithium Metal Anodes

**FIGURE 1:**
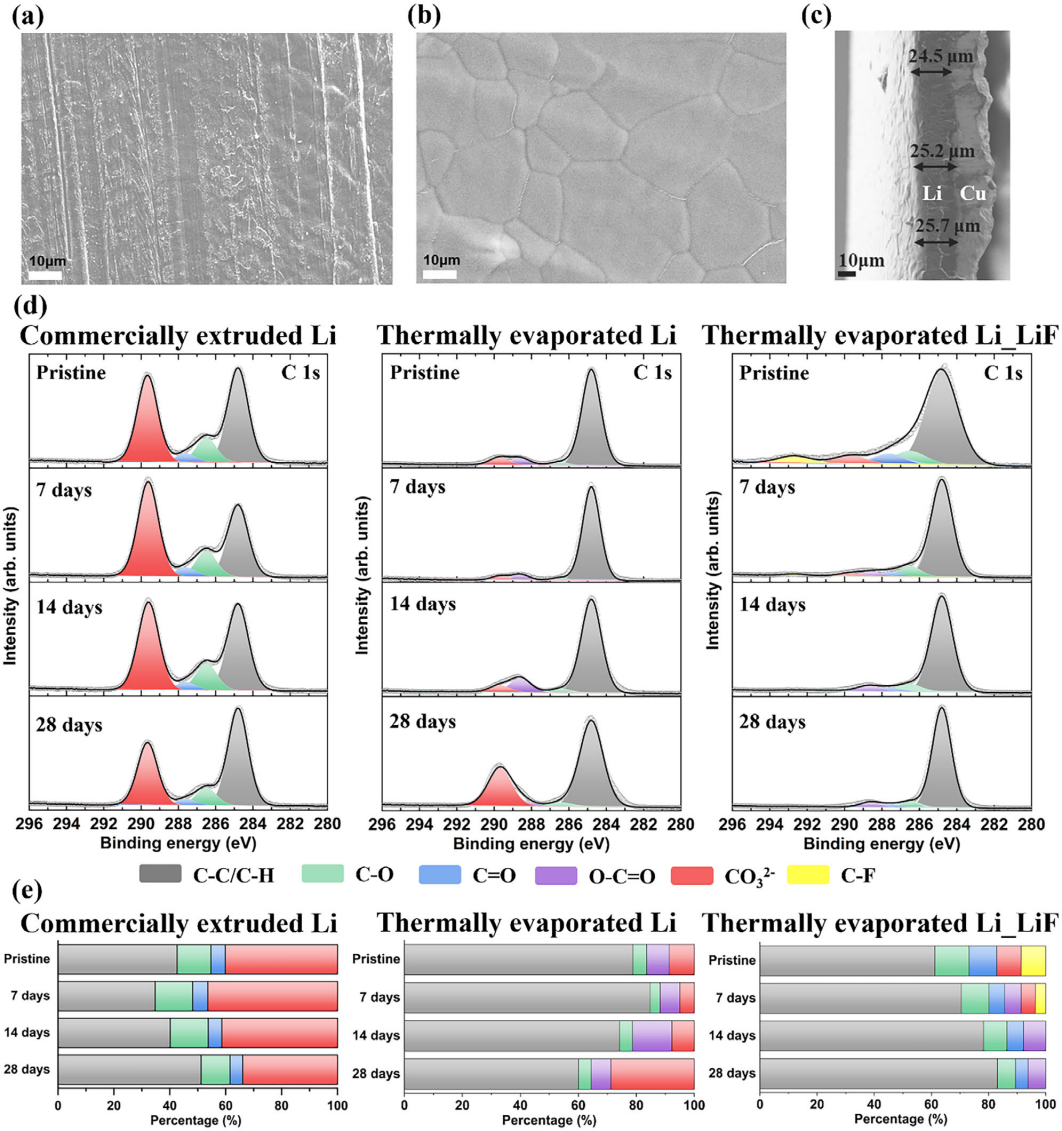
Comparison of different types of lithium metal: commercially extruded lithium, thermally evaporated lithium, and thermally evaporated lithium passivated by 65 nm LiF. Top view SEM images performed on (a) commercially extruded and (b) thermally evaporated lithium. (c) Cross‐sectional SEM image of 25 µm thermally evaporated lithium on Cu foil. (d) C1s XPS spectra performed after storage in Ar‐filled glovebox for 7, 14, 28 days. (e) Percentage distribution of various components in the C 1s spectra.

The top‐view scanning electron microscopy (SEM) image of the 50 µm commercially extruded lithium (Figure 1a) reveals a markedly rough surface morphology characterized by wrinkles, grooves and shallow hollows. These features originate from the mechanically induced deformation and the formation of a thick native passivation layer during the industrial rolling, calendaring and manufacturing processes. In contrast, the thermally evaporated lithium (Figure 1b) exhibits a much smoother and homogeneous surface, with well‐defined grain boundaries. These features of microstructure are consistent with previous observations reported by Ingenito et al. [15], who demonstrated that vapor‐phase deposition produces highly uniform lithium layers with minimal surface contamination. The evaporated lithium metal films were cross‐sectioned and subsequently imaged by SEM. As illustrated for the 25 µm (Figure 1c) and 50 µm (Figure S1) samples, the evaporation process provides excellent control over film thickness and uniformity. The cross‐sectional SEM images further confirm that the lithium film is densely and conformally deposited onto a 20 µm thick copper current collector. The film shows a compact, pore‐free microstructure and maintains intimate interfacial contact with the Cu substrate, indicating both high deposition uniformity and strong mechanical integrity.

X‐ray photoelectron spectroscopy (XPS) (Figure 1d) was employed to examine the surface chemical composition of the different lithium metal types and their evolution during storage in the glovebox. At the pristine stage, the C 1s spectra of commercially extruded lithium exhibited characteristic peaks corresponding to C‐C/C‐H, C‐O, C = O, CO_3_^2−^ species with binding energies of 284.8, 286.5, 287.6, 289.7 eV, respectively. The surface is dominated by the C‐C/C‐H and carbonate (CO_3_^2−^) species originated from the reaction of metallic lithium with CO_2_ and moisture during processing and storage in the dry room, which is difficult to avoid and is commonly observed for commercially extruded lithium [15, 19, 20]. In contrast, thermally evaporated lithium, deposited under high vacuum (10^−7^ mbar), showed significantly diminished oxidized carbon signals, confirming the formation of a fresh and minimally contaminated lithium surface.

When the thermally evaporated lithium was further coated with a 65 nm lithium fluoride (LiF) passivation layer and measured in situ, a new peak emerged at a high binding energy of 292.7 eV, attributed to the formation of C‐F species. The XPS of the F 1s core level (Figure S2) demonstrated the presence of an intense LiF peak at 685.1 eV and a minor C‐F peak at 688.2 eV.

After initial characterization at pristine stage, all samples were stored inside an Ar‐filled glovebox and re‐measured after 7, 14, 28 days. The C 1s spectra of extruded lithium remains largely unchanged and is always dominated by C‐C/C‐H and CO_3_^2−^ species over time, reflecting the stability of its native passivation layer. Conversely, thermally evaporated lithium showed an increase of C‐C/C‐H content in the first 7 days and then experienced a progressive increase in O‐C = O (288.6 eV) and CO_3_^2−^ peaks, indicating gradual surface oxidation in the absence of a stable passivation layer. Remarkably, the LiF‐coated thermally evaporated lithium exhibited excellent chemical stability, with no significant emergence or growth of oxidized carbon species during the entire storage period.

Quantitative analysis of the C 1s spectra (Figure 1e) further highlights the distinct surface chemical evolution of the three lithium types. For commercially extruded lithium, the carbonate fraction was 40% at the pristine stage and 34% after 28 days. The minor variation was attributed to the non‐uniform surface morphology of extruded Li (Figure 1a), while the overall stability originated from the passivation effect of the native Li_2_CO_3_ layer. In comparison, thermally evaporate lithium initially exhibited only 8.7% carbonate, which increased substantially to 28.8% after 28 days, indicating gradual surface oxidation. For evaporated lithium passivated with 65 nm of LiF layer, the initial carbonate fraction was 8.5%, similar to that of uncoated evaporated lithium, with an additional 8.7% assigned to C‐F species. Upon storage, mainly the hydrocarbon C‐C/C‐H component increased, while the formation of carbon oxidized species was much less pronounced. No CO_3_^2−^ signal was detected after 14 and 28 days, indicating that the LiF layer effectively prevented carbonate formation.

Here, it should be emphasized that the pristine stage of the LiF‐passivated lithium was measured in‐situ without breaking the vacuum, in contrast to the other two lithium films, which were exposed to the glovebox environment prior to measurement. The comparison of survey scans and the corresponding atomic percentages of C, O, Li, F are summarized in Figures S3 and S4. For commercially extruded lithium, the atomic percentages of C and O remained stable during storage, fluctuating around 26% and 36%, respectively. For thermally evaporated lithium, the C percentage was as high as 46% at the pristine stage owing to the presence of fresh Li without a passivation layer. This value increased to 51% during the first 7 days, followed by a substantial rise in O percentage to 34% after 28 days, consistent with the surface oxidation trend observed in C 1s spectra (Figure 1d,e). In contrast, for LiF‐passivated evaporated lithium, the deposited LiF layer attenuated the signal from the native surface. Additionally, because XPS measurement was performed in‐situ under ultra‐high vacuum transfer, adventitious carbon did not evolve, resulting in a low initial C percentage (3%). Consequently, the full width at half maximum (FWHM) of C 1s spectra at pristine stage was relatively broad. After storage in the glovebox, the C percentage increased to 28% after 28 days, consistent with the increased C‐C/C‐H signal. This adventitious carbon accumulation progressively covered the initial lithium surface, leading to a continuous decrease in the detected C‐F and CO_3_^2−^ species.

The evolution of O 1s, Li 1s, and F 1s spectra is summarized in Figures S5‐S7. For thermally evaporated lithium, the increased binding energies of the O 1s spectra (from 531 to 531.6 eV) and Li 1s spectra (from 54.7 to 55 eV) after 28 days are consistent with the gradual surface oxidation observed in the C 1s spectra (Figure 1d). In the case of LiF‐passivated evaporated lithium, the decrease in O 1s binding energy (from 532.1 to 531.7 eV) and the attenuation of the C–F component in the F 1s spectra are attributed to the accumulation of adventitious carbon during storage. The higher binding energy of the Li 1s spectra (55.7 eV) confirms the formation of LiF.

To summarize, commercially extruded lithium contains a substantial amount of stable Li_2_CO_3_ from the beginning. Thermally evaporated lithium exhibits low initial oxidation but undergoes gradual degradation during storage. In contrast, LiF‐passivated evaporated lithium demonstrates excellent long‐term surface chemical stability and prohibits the formation of Li_2_CO_3_. This protective effect originates from the thermodynamical stability of LiF [21], owing to its very low Gibbs free energy of formation (−588 KJ mol^−1^) [22], which effectively suppressed surface reactions.

### 2.2 Electrochemical Cycling Performance as a Function of PVD Lithium Thickness

#### 2.2.1 Li|LPSCl|Li Symmetric Cells

Thermally evaporated lithium films with varying thicknesses were employed in Li|Li symmetric cells to investigate the influence of lithium reservoir size on the cycling behavior and interfacial stability. Four lithium films with nominal thicknesses of 12.5, 25, 37.5, and 50 µm, denoted as Li_12.5 µm, Li_25 µm, Li_37.5 µm, and Li_50 µm, respectively, were prepared for systematic comparison. A stack pressure of 20 MPa was applied during electrochemical cycling. This value was selected based on our previous work [23], which demonstrated that 20 MPa provides sufficient interfacial contact while suppressing Li dendrite formation, thereby ensuring excellent cycling reproducibility and reliability in ASSLMBs.

**FIGURE 2:**
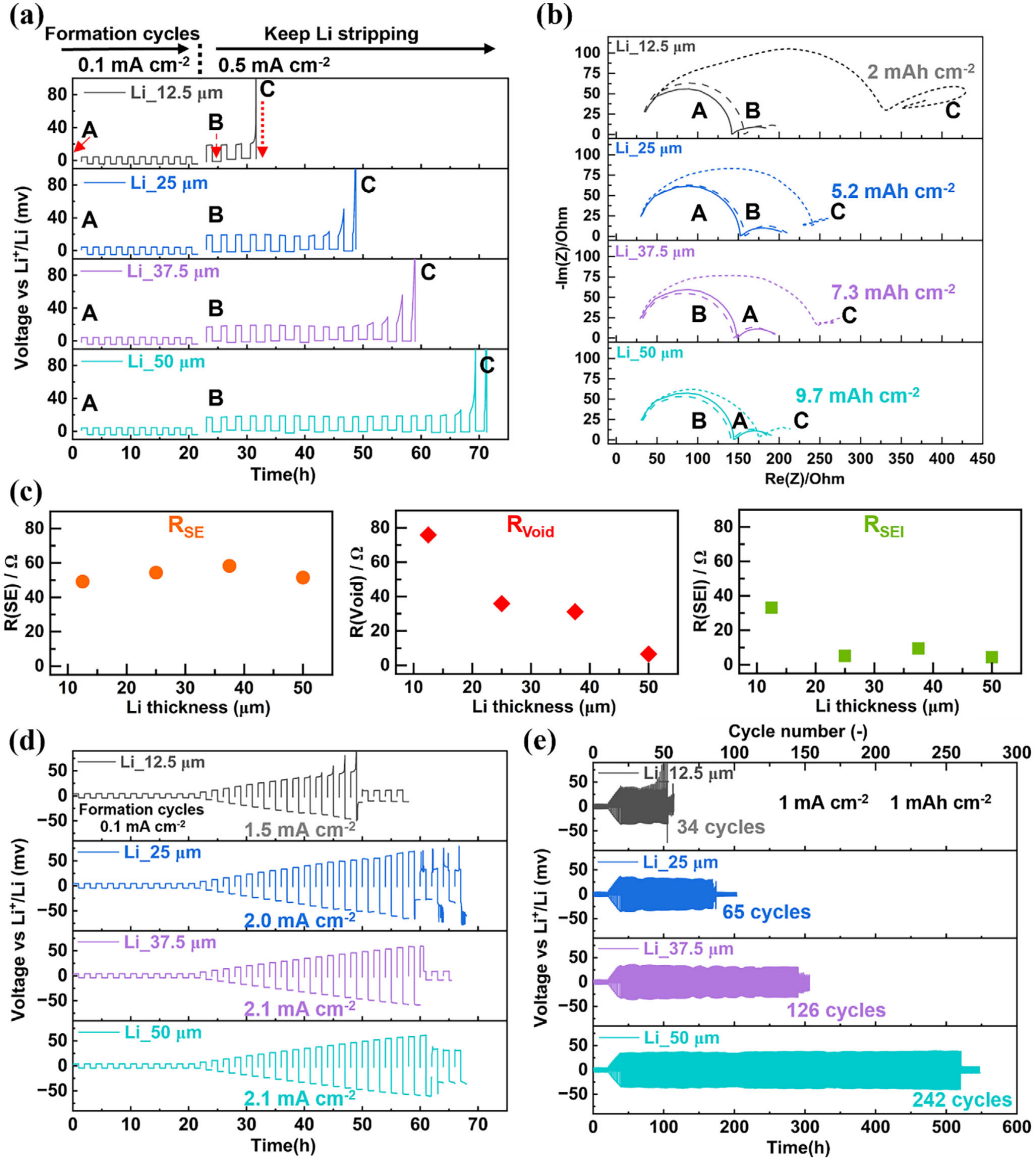
Cycling performance of thermally evaporated lithium with different thicknesses (12.5, 25, 37.5, 50 µm). (a) Voltage profiles during continuous lithium stripping. (b) Corresponding EIS spectra recorded at testing points A, B, and C in (a). (c) DRT analysis corresponding to the resistance of solid electrolyte (SE), void, and solid electrolyte interphase (SEI) at testing point C. (d) Critical current density measurements. (e) Long‐term cycling performance at 1 mA cm^−2^ and 1 mAh cm^−2^.

As shown in Figure 2a, each cell first underwent a formation protocol consisting of lithium plating and stripping at 0.1 mA cm^−2^ with an areal capacity of 0.1 mAh cm^−2^. After formation cycles, continuous lithium stripping was performed at 0.5 mA cm^−2^ until the cell voltage reached the cut‐off value of 100 mV, corresponding to complete depletion of electrochemically active lithium. The voltage discontinuities observed in the cycling curves originated from the impedance measurements integrated into the cycling protocol. At predefined intervals of 1 h during lithium stripping, the cycling was temporarily paused to perform electrochemical impedance spectroscopy (EIS), allowing real time monitoring of interfacial resistance evolution.

Depending on the initial lithium thickness, the extracted (stripped) areal capacities were 2, 5.2, 7.3, 9.7 mAh cm^−2^, corresponding to a lithium equivalent thickness of 9.7, 25.2, 35.4, 47.1 µm for Li_12.5 µm, Li_25 µm, Li_37.5 µm, and Li_50 µm samples, respectively. These values demonstrate that a substantial fraction of thermally evaporated lithium is electrochemically accessible. As illustrated in Figure S8, 77% of the nominal lithium thickness was actively stripped at 0.5 mA cm^−2^ for Li_12.5 µm film. Thicker lithium films (25 to 50 µm) exhibited an even larger fraction of active lithium, exceeding 94%.

EIS spectra were recorded at three characteristic states (Figure 2b): point A (before cycles), B (after the first lithium stripping at 0.5 mA cm^−2^, equivalent to 2.4 µm Li), and C (after reaching 100 mV), to evaluate the evolution of void formation and SEI growth at the Li|LPSCl interface. For the Li_12.5 µm films, the impedance increased from point A to B, indicating the onset of interfacial degradation. In contrast, for thicker films (25 to 50 µm), the impedance at point B decreased, with Li_37.5 µm and Li_50 µm even exhibiting lower resistance than their initial states. This improvement can be attributed to the applied cycling pressure of 20 MPa and the use of formation cycles, both of which help mitigate interfacial imperfections [23]. Additionally, the fraction of stripped Li at point B (4.8%) was very small relative to the large fraction of active lithium (94%) for Li_50 µm. Consequently, the thicker lithium reservoir is able to maintain intimate interfacial contact and suppress void formation. By comparison, stripping the same absolute amount of lithium from the thin Li_12.5 µm electrode corresponds to a much larger relative fraction (19.2%) to its active lithium (77%), which is sufficient to induce void formation and increase interfacial resistance [24].

After complete lithium depleting (point C), the EIS spectra displayed pronounced thickness‐dependent differences. The semi‐circle feature (∼ 1 MHz – 100 Hz) associated with interfacial resistance increased substantially for the Li_12.5 µm while decreased progressively with increasing lithium thickness. To quantitatively assess impedance evolution, a Distribution of Relaxation Times (DRT) analysis was applied to the EIS spectra at point C. As shown in Figure S9, features at time constants smaller than ∼10^−7^ s correspond to the solid electrolyte pellet resistance (R_SE_), while those in the 10^−5^ and 10^−2^ s ranges are attributed to the solid electrolyte interphase resistance (R_SEI_). The intermediate timescale represents void‐related resistance (R_Void_), in agreement with previous literature [25, 26, 27]. As summarized in Figure 2c, R_SE_ remained nearly constant across all film thicknesses (49.2 Ω for Li_12.5 µm, and 51.5 Ω for Li_50 µm), confirming uniform SE pellet quality. In contrast, R_Void_ showed a strong dependence on lithium thickness. Notably, R_Void_ for Li_12.5 µm was as high as 75.9 Ω, demonstrating that insufficient lithium reservoir leads to local depletion and severe voids formation [28]. Increasing lithium thickness markedly reduced R_Void_, from 35.8 Ω for Li_25 µm to 6.6 Ω for Li_50 µm. The same trend was observed for R_SEI_, which decreased from 33.1 Ω for Li_12.5 µm to 4.3 Ω for Li_50 µm. These results collectively demonstrate that thicker lithium films enhance interfacial stability by providing sufficient lithium to fill voids during cycling and maintain contact with the solid electrolyte [29].

Critical current density (CCD) and long‐term cycling tests were further performed to assess electrochemical robustness. As shown in Figure 2d, the CCD increased from 1.5 mA cm^−2^ for Li_12.5 µm to 2 mA cm^−2^ for Li_25 µm, with marginal improvement for thicker films (2.1 mA cm^−2^ for Li_37.5 µm, and Li_50 µm). Long‐term cycling at 1 mA cm^−2^ and 1 mAh cm^−2^ (Figure 2e) showed that the cycle life extended markedly with increasing lithium thickness, from 34 cycles for Li_12.5 µm to 242 cycles for Li_50 µm. Notably, only Li_12.5 µm exhibited a significant overpotential increase prior to short circuit, consistent with void‐induced degradation followed by dendrite initiation, as reported by Janek et al. [30]. In contrast, thicker lithium electrodes maintained stable voltage profiles during cycling, with eventual failure dominated by crack formation and dendrite penetration, leading to sudden voltage drops [4, 31]. Overall, thin Li 12.5 µm suffered from severe void formation and high interfacial resistance, while increasing lithium thickness effectively mitigated these issues, resulting in higher CCDs and improved cycling stability.

#### 2.2.2 NCM811|LPSCl|Li Full Cells

**FIGURE 3:**
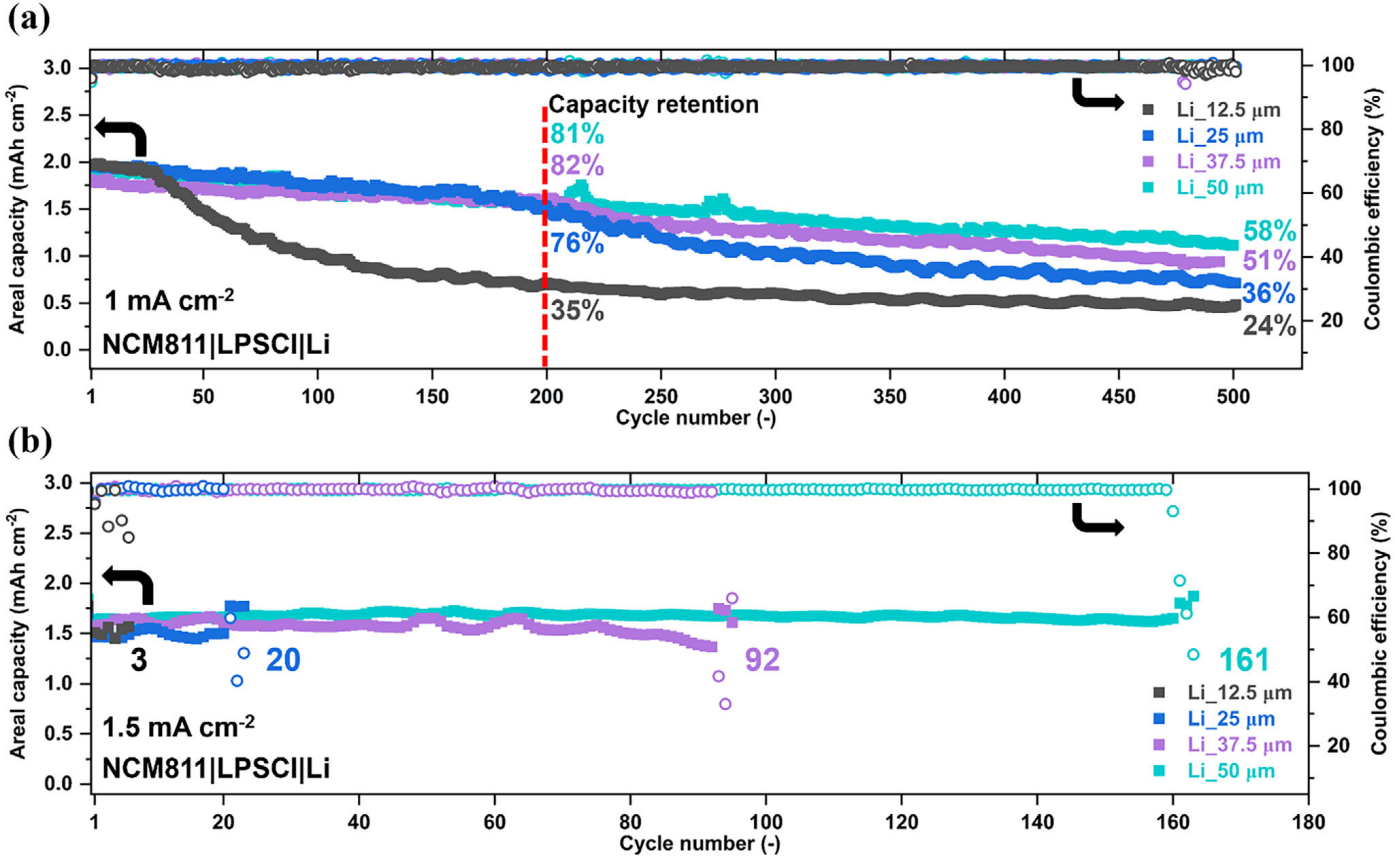
Long term cycling performance of NCM811|LPSCl|Li full cells employing thermally evaporated lithium metal anodes with different thicknesses (12.5, 25, 37.5, 50 µm), at current density of (a) 1 mA cm^−2^, (b) 1.5 mA cm^−2^.

The full cell cycling performance was systematically evaluated employing LiNi_0.8_Co_0.1_Mn_0.1_O_2_ (NCM811) cathodes and thermally evaporated lithium anodes of varying thicknesses. At a current density of 1 mA cm^−2^ (Figure 3a), all cells delivered a similar areal capacity of approximately 1.9 mAh cm^−2^, corresponding to the plating/stripping of 9.2 µm of lithium equivalent thickness. Despite similar initial capacities, the long‐term cycling behavior strongly depended on the anode thickness. After 25 cycles, the thinnest Li_12.5 µm electrode exhibited pronounced capacity fading, retaining only 35% of its initial capacity after 200 cycles. In contrast, Li_25 µm and Li_50 µm electrodes showed markedly improved stability, maintaining 76% and 81% capacity retention after 200 cycles, respectively. Beyond 200 cycles, the capacity of Li_12.5 µm remained relatively constant due to the limited thickness of stripped lithium (∼ 3.2 µm), whereas Li_25 µm continued to fade, reaching 36% retention after 500 cycles. The Li_50 µm cell exhibited the best durability, retaining 58% capacity after 500 cycles.

The corresponding voltage profiles of charge and discharge (Figure S10) further highlight the strong thickness‐dependent behavior. All cells exhibited an identical onset charge voltage of 3.71 V. After 500 cycles, this value increased to 4.04 V for Li_12.5 µm, indicating growing interfacial resistance, whereas Li_50 µm maintained a moderate voltage of 3.8 V. Furthermore, the thin lithium anodes displayed a rapid voltage drop near the end of discharge, in contrast to the smoother and more continuous lithium stripping behavior observed for Li_50 µm. These results confirm that thinner lithium anodes experience limited available electrochemically active lithium caused by the poor interfacial stability, thereby leading to larger overpotentials during cycling.

At a high current density of 1.5 mA cm^−2^ and an areal capacity of ∼1.6 mAh cm^−2^ (Figure 3b), the detrimental effect of limited lithium inventory became more pronounced. The Li_12.5 µm cell experienced an early short circuit after only three cycles, whereas the Li_25 µm and Li_50 µm cells achieved extended lifetimes of 20 and 161 cycles, respectively.

These thickness dependency behaviors in NCM811|Li full cells are consistent with those observed in Li|Li symmetric cells (Figure 2). Thin lithium anodes, particularly Li_12.5 µm, suffered from void formation and high interfacial resistance with the SE, resulting in interfacial instability, large overpotentials, and premature failure. Although thicker thermally evaporated lithium anodes improved cycling stability and extended lifespan, gradual capacity fading and limited high‐rate performance were still observed.

### 2.3 Electrochemical Cycling Performance of LiF Surface‐Passivated PVD Lithium Anodes

To address the interfacial instability while preserving high energy density with minimal lithium excess, Li_25 µm anode was selected as the baseline for the subsequent incorporation of a LiF passivation layer deposited by electron‐beam evaporation. The LiF deposition process followed our previous work [32], in which an evaporation rate of 6.5 nm min^−1^ was established.

The EDX mapping of F Kα (Figure S11a‐c) revealed a progressive increase in fluorine areal coverage from 79% for a 40 nm LiF thick layer to 89% and 94% for 65 and 100 nm LiF layers, respectively, indicating increasingly complete fluorine coverage on the Li metal surface with increasing LiF thickness. However, conventional EDX maps are discretized images in which the elemental intensity is represented by spatially distributed photon counts, making quantitative evaluation of local homogeneity difficult due to signal fluctuations and noise.

To further evaluate the spatial uniformity of the LiF layer, Gaussian kernel density (GKD) mapping was applied to the EDX results to reconstruct a continuous fluorine concentration field that more accurately reflects the elemental distribution. The detailed methodology of the GKD analysis has been reported in our previous work [33]. In this approach, the photon count data are incorporated into a 2D density map (Figure S11d‐f), enabling visualization of the LiF spatial distribution and local fluctuation of the F Kα signal intensity.

For comparison, the density values were normalized by the median F Kα signal intensity on the Li metal anodes, which effectively removes the information on the absolute intensity but displays the inhomogeneity with a high sensitivity. Accordingly, blue regions represent lower local fluorine density, white regions correspond to intermediate density, and red‐to‐yellow regions indicate fluorine‐enriched domains.

As shown in Figure S11d‐f, the fluorine distribution becomes progressively more homogeneous with increasing LiF thickness. The 40 nm LiF layer exhibits pronounced local fluctuations with numerous interconnected red/yellow and blue domains, indicating significant spatial heterogeneity of fluorine distribution. In contrast, the 65 nm LiF layer shows substantially reduced color contrast and a more continuous elemental distribution, suggesting markedly improved surface uniformity. Compared with the 65 nm LiF layer, the 100 nm LiF layer exhibits only a modest additional reduction in color contrast, with both maps already dominated by relatively uniform light regions and only sparse high‐ or low‐density domains. This trend is further supported by quantitative GKD analysis. Specifically, the coefficient of variation decreased from ∼40.3% for the 40 nm LiF layer to ∼15.6% and ∼14.3% for the 65 and 100 nm LiF layers, respectively. These results indicate a significant improvement in fluorine homogeneity from 40 to 65 nm LiF, whereas the additional homogenization achieved at 100 nm is comparatively limited.

Overall, the combined EDX and GKD analyses confirm that increasing the LiF thickness leads to improved fluorine coverage and a substantially more homogeneous LiF layer on the Li metal surface. In particular, the 65 and 100 nm LiF passivation layers exhibit highly uniform fluorine distributions with low spatial variance, supporting the formation of continuous and well‐distributed protective LiF interphases on the Li metal anodes.

**FIGURE 4:**
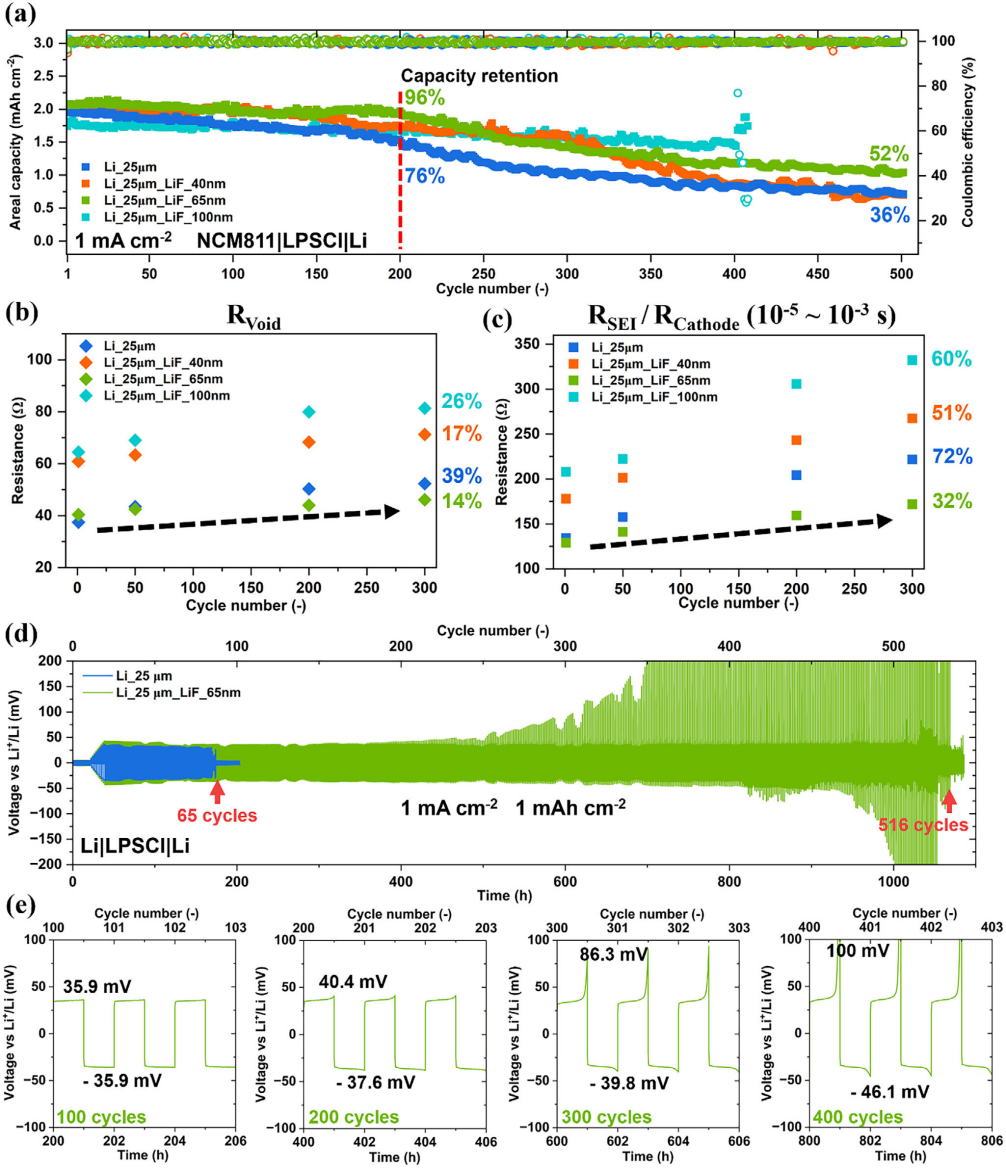
Comparison of thermally evaporated Li (25 µm thick) anodes coated with LiF layers of varying thicknesses. (a) Long‐term galvanostatic cycling of NCM811|LPSCl|Li full cells at 1 mA cm^−2^. DRT analysis corresponding to the evolution of resistance with cycle number, (b) void resistance (R_Void_), and (c) combined SEI and cathode resistance (R_SEI_ / RCathode) within time regime from 10^−5^ s to 10^−3^ s, in NCM811|LPSCl|Li full cells. (d) Long‐term galvanostatic cycling of Li|LPSCl|Li symmetric cells at 1 mA cm^−2^ and 1 mAh cm^−2^. (e) Zoom‐in voltage profiles of LiF‐passivated lithium corresponding to cycles 100, 200, 300, and 400 in (d).

The long‐term cycling of NCM811|Li full cells at 1 mA cm^−2^ (Figure 4a), demonstrated that the 65 nm LiF coating significantly enhanced capacity retention, increasing from 76% for pristine evaporated Li_25 µm to 96% after 200 cycles. This value even surpassed that of the pristine thicker Li_50 µm electrode (81%). After 500 cycles, the 65 nm coated Li_25 µm retained 52% capacity, comparable to the pristine thicker Li_50 µm (58%, Figure 3a). In contrast, the 40 nm LiF coating degraded after 100 cycles, reaching 37% capacity retention after 500 cycles, similar to that of pristine Li_25 µm (36%). For the 100 nm LiF coated Li, despite a lower initial areal capacity (1.74 mAh cm^−2^) compared to pristine lithium (1.95 mAh cm^−2^) and the 65 nm LiF‐coated Li (2.08 mAh cm^−2^), the cell short‐circuited after 400 cycles. Altogether, these results show that a 65 nm LiF passivation layer provides the most balanced performance, delivering both the highest initial areal capacity and the most stable long‐term cycling performance, particularly within the first 200 cycles.

EIS combined with DRT analysis performed on NCM811|LPSCl|Li full cell was recorded after the first, 50th, 200th, and 300th cycles, as shown in Figure S12. To accurately assign the time constants to the corresponding resistance contributions, the EIS measurement was performed using three‐electrode cells, enabling the separation of the resistances at the anode and cathode side. Detailed procedures for cell assembly, in situ lithiation of gold‐plated tungsten wire (denoted Au/W wire) used as a reference electrode, and electrochemical measurements are described in the experiment section and our reported study [34]. The corresponding DRT patterns obtained between the NCM811 working electrode (WE) and reference electrode (RE), the lithium metal counter electrode (CE) and RE, and between the WE and CE are shown in Figure S13. Six main peaks are observed in the time region spanning from 5.10^−6^ to 0.5 s. The resistance associated with void (R_Void_) appears in a similar region between 10^−6^ to 10^−5^ s as that observed in Li|Li symmetric cells. From 10^−5^ to 5.10^−2^ s, the DRT pattern of the WE‐CE is formed by the superposition of one pronounced peak in the WE‐RE signal and three less prominent peaks in the CE‐RE signal, corresponding to the cathode resistance (R_Cathode_) and SEI resistance at the anode (R_SEI_), respectively. At a time domain of approximately 10^−1^ s, a peak emerges in the WE‐RE signal, whereas no peak is observed in the CE‐RE signal, indicating that R_Cathode_ dominates the DRT pattern of the WE‐RE signal in this region.

Based on these assignments enabled by the three‐electrode cell, the DRT pattern of NCM811|LPSCl|Li full cell (Figure S12) between 10^−6^ to 10^−1^ s can be divided into three distinct regions: R_Void_ (10^−6^ to 10^−5^ s), combined SEI and cathode resistance (R_SEI_ / R_Cathode_) (10^−5^ to 5.10^−2^ s), and R_Cathode_ (∼10^−1^ s). The relative ordering of these resistance contributions is consistent with time‐domain assignments reported by Coskun et al. and Zhang et al. for sulfide‐based SE/Li metal systems, confirming that the interfacial resistance between the anode and SE manifests at higher frequencies than cathode‐related processes [35, 36, 37]. Furthermore, this analysis distinguishes regions with overlapping resistance contributions from those with dominant resistance.

From the evolution of R_Cathode_ (∼10^−1^ s) (Figure S14), the resistance increased by 66% after 300 cycles, rising from 20.8 Ω to 34.7 Ω for the cell with pristine lithium. This is comparable to the 60% increase observed for lithium with a 65 nm LiF layer (from 22.3 Ω to 35.7 Ω). Excluding the larger 75% increase corresponding to the thicker LiF layer (100 nm), which is attributed to its poor ionic transport, R_Cathode_ (∼10^−1^ s) exhibits similar initial values and growth trends across cells with different anode materials. This behavior is reasonable, as the cathode material and preparation process are identical in all cells, further demonstrating the reliability of the time‐domain assignments derived from the three‐electrode cell.

The evolution of R_Void_ (Figure 4b) demonstrates that the 65 nm LiF layer effectively maintains interfacial contact between the lithium metal anode and SE, suppressing void formation. For the pristine Li_25 µm anode, R_Void_ increased by 39%, from 37.5 Ω to 52.3 Ω after 300 cycles. In contrast, with the 65 nm LiF coating, the increase was significantly mitigated to 14%, rising from 40.4 Ω to 46.1 Ω.

To investigate the evolution of R_SEI_, it is essential to allocate an appropriate time regime in which R_SEI_ predominantly contributes to the evolution of total resistance. In the overlapping region of combined SEI and cathode resistance (R_SEI_ / R_Cathode_) (10^−5^ to 5.10^−2^ s) shown in Figure S12, three peaks (R_1_, R_2_, R_3_) are observed, with the primary differences among lithium metal anodes appearing in R_1_ and R_2_. Additionally, Figure S13 shows that the resistance corresponding to the R_3_ (∼10^−2^ s) is dominated by R_Cathode_ with a peak value approximately five times higher than that of R_SEI_ in this region. Consequently, the time‐region including R_1_ and R_2_, from 10^−5^ s to 10^−3^ s, is selected to compare the evolution of R_SEI_. Although the resistance in this region still contains contributions from both R_SEI_ and R_Cathode_, the similar R_Cathode_ growth (Figure S14) observed across all cells allows the evolution trend of the resistance to be attributed to R_SEI_.

From Figure 4c, R_SEI_ (10^−5^ to 10^−3^ s) of the pristine Li_25 µm increased substantially by 72%, from 134.1 Ω to 221.7 Ω, whereas the increase with 65 nm LiF coating was limited to 32%, from 129 Ω to 171.7 Ω, indicating markedly suppressed interfacial degradation at the anode. However, both insufficient LiF coverage and excessive LiF thickness adversely affected interfacial stability. Owing to the limited protective capability of the 40 nm LiF layer and the poor ionic transport through the thicker 100 nm LiF layer resulting from its intrinsically low ionic conductivity [38, 39], both R_Void_ and R_SEI_ exhibited higher initial resistances and more pronounced increases during cycling compared with the optimized 65 nm LiF layer.

Despite the improvements in interfacial stability between the lithium metal anode and SE with optimized LiF passivation, long‐term degradation was still evident. As shown in Figure 4d, although the lifetime of Li|Li symmetric cells operated at 1 mA cm^−2^ and 1 mAh cm^−2^ extended markedly from 65 cycles for pristine Li_25 µm to 516 cycles, the overpotential gradually became significant. From Figure 4e, the absolute voltage at the 100th cycle was 35.9 mV after both stripping and plating. After 200 and 300 cycles, the post‐stripping voltage increased to 40.4 and 86.3 mV, respectively, whereas the post‐plating values rose more moderately to 37.6 and 39.8 mV. By the 400th cycle, the post‐stripping voltage exceeded 100 mV, and the post‐plating voltage reached 46.1 mV. Ultimately, the voltage surpassed 100 mV for both stripping and plating, leading to short circuits at the 516th cycle.

These results indicate that the increase in overpotential on the lithium electrode undergoing stripping during the first cycle becomes more severe upon long cycling, due to void formation and the accumulation of inactive lithium from inhomogeneous stripping. The progressive rise in overpotential in Li|Li symmetric cells with cycled lithium equivalent thickness of 4.85 µm on both electrodes (stripping‐initiated) correlates well with the capacity fading observed in full cells with cycled lithium of 9.7 µm on anodes (plating‐initiated), both of which begin to degrade after 200 cycles.

**FIGURE 5:**
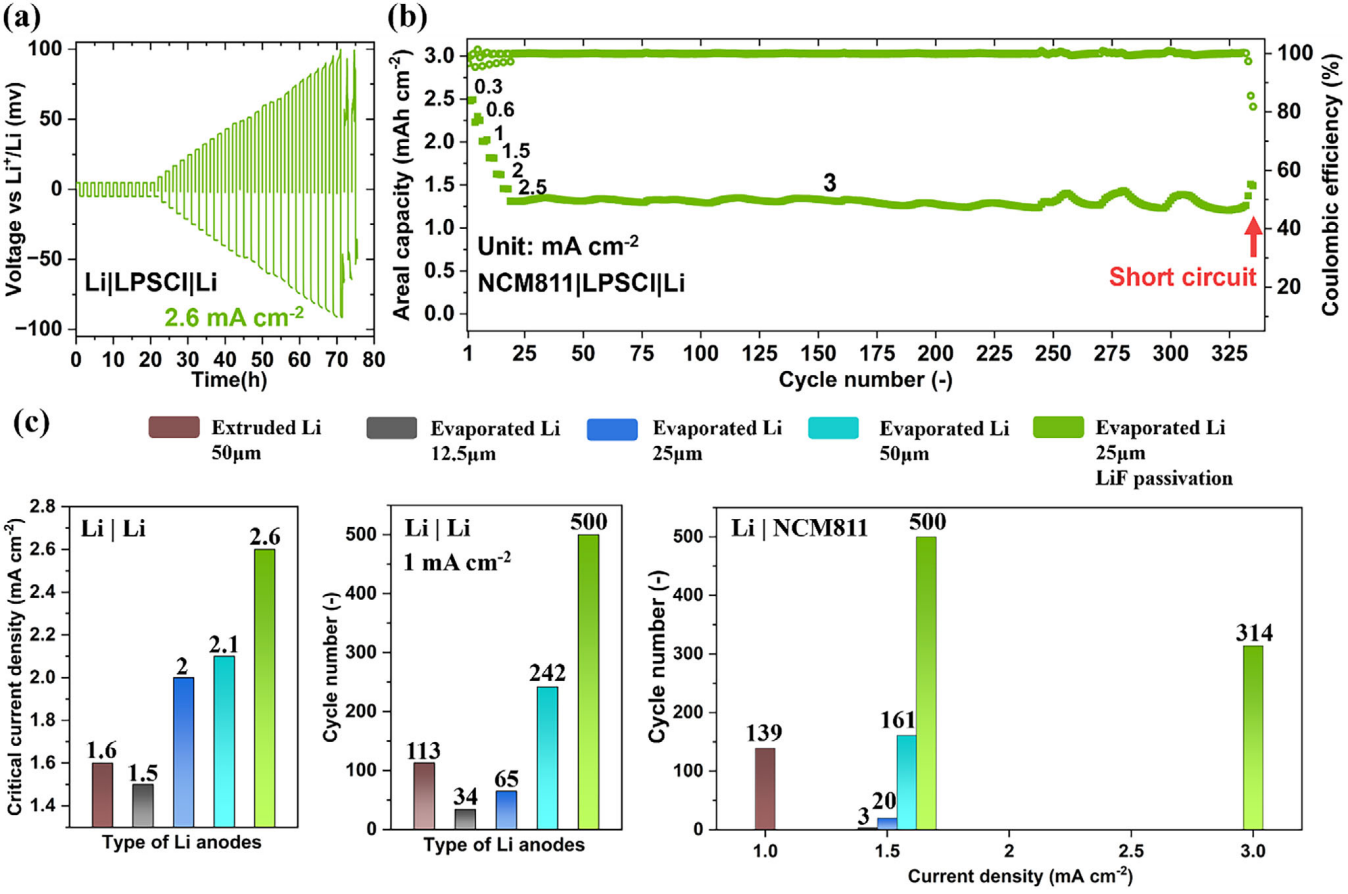
Cycling performance of thermally evaporated Li (25 µm thick) anodes coated with a 65 nm LiF passivation layer. (a) Critical current density test of Li|Li symmetric cell, (b) Long‐term cycling of NCM811|LPSCl|Li full cell at 3 mA cm^−2^. (c) Performance comparison of Li|Li symmetric cells and full cells employing different types of Li anodes, the data corresponding to extruded Li (50 µm) is from Ref. [32], rights and permissions are provided by the article's Creative Common license.

The cycling performance of 25 µm thermally evaporated lithium anodes with an optimized 65 nm LiF passivation layer was further evaluated under harsh conditions. In Li|Li symmetric cells (Figure 5a), the CCD increased significantly from 2 mA cm^−2^ for pristine lithium (Figure 2d) to 2.6 mA cm^−2^ after LiF passivation. For NCM811|Li full cells, the cycling lifespan was extended dramatically from 20 to 500 cycles at a current density of 1.5 mA cm^−2^ with a capacity retention of 54% (Figure S15). As shown in Figure 5b, when cycled with progressively increasing current densities from 0.3 to 3 mA cm^−2^ (3 cycles per step) and subsequently sustained at 3 mA cm^−2^, the cell maintained stable operation with an initial areal capacity of 1.3 mAh cm^−2^ for over 300 cycles before short circuiting. Together with the voltage profiles shown in Figure S16, the capacity retention reached 91% after 250 cycles, owing to the relatively low areal capacity, which corresponds to an equivalent lithium thickness of 6.3 µm. In contrast, the higher areal capacity of 2 mAh cm^−2^ (9.7 µm in Figure 4a) at 1 mA cm^−2^ resulted in a lower capacity retention of 79% after 250 cycles, highlighting the detrimental effect of stripping a large fraction of the available lithium.

### 2.4 Discussion and Comparison

Figure 5c compares the cycling performance of both Li|Li symmetric cells and NCM811|Li full cells employing different types of lithium anodes with varying thicknesses. The commercially extruded Li (50 µm) exhibited relatively weak performance, delivering a CCD of 1.6 mA cm^−2^ and a cycle life of 113 cycles at 1 mA cm^−2^ in Li|Li symmetric cells. In contrast, the thermally evaporated Li (50 µm) demonstrated substantially improved performance, with the CCD increasing from 1.6 to 2.1 mA cm^−2^ and the cycle life extending from 113 to 242 at 1 mA cm^−2^ in symmetric cells. Consistent with the symmetric cells results, the cycling stability of NCM811|Li full cells also improved markedly, increasing to 161 cycles at 1.5 mA cm^−2^ when the anodes were replaced from extruded Li (50 µm) to evaporated Li (50 µm).

However, reducing the thickness of the evaporated Li to 25 µm resulted in inferior long‐term cycling performance, with the full cells sustaining 20 cycles at 1.5 mA cm^−2^. This degradation is attributed to enhanced void formation and increased interfacial resistance associated with thinner Li electrodes, as discussed in Figure 2. By introducing an optimized LiF passivation layer deposited onto the evaporated Li (25 µm), the performance was significantly enhanced. The modified electrode achieved a CCD of 2.6 mA cm^−2^ and sustained stable cycling for 500 cycles at 1 mA cm^−2^ in Li|Li symmetric cells, while enabling 314 cycles at a high current density of 3 mA cm^−2^ in NCM811|Li full cells.

Moreover, as summarized in Table S1, the cycling performance achieved in this work with 25 µm PVD Li anode is comparable to, and in several cases exceeds, previously reported results in the selected literature, even when the lithium has twice the thickness (50 µm), particularly in terms of long‐term cycling stability, sustained capacity retention, and operation under relatively high current density and areal capacity conditions. To ensure a meaningful comparison, the selected studies were limited to systems employing Li_6_PS_5_Cl‐based solid electrolytes, thin lithium metal anodes with thicknesses of approximately 50 µm or below, and NCM‐based cathodes. These results demonstrate the effectiveness of the LiF‐passivated thermally evaporated Li in enhancing the electrochemical performance of ASSLMBs.

**FIGURE 6:**
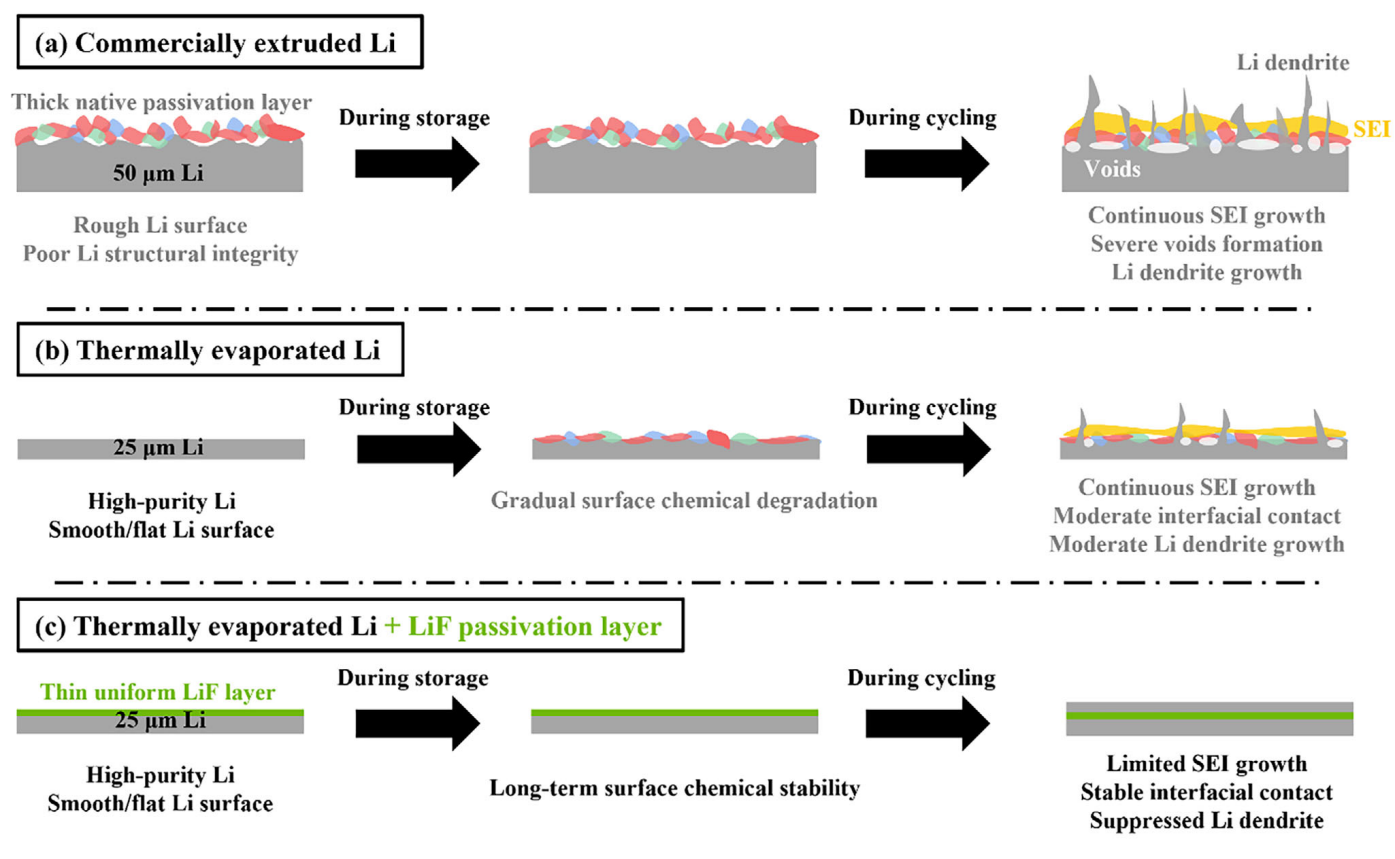
Schematic illustrations of different types of lithium metal anodes during storage and electrochemical cycling, (a) commercially extruded Li, (b) thermally evaporated Li, (c) thermally evaporated Li combined with a LiF passivation layer.

Figure 6 schematically summarizes the working principles and advantages of thermally evaporated lithium metal anodes combined with a LiF passivation layer during storage and electrochemical cycling. Commercially extruded Li exhibits a rough surface morphology and poor structural uniformity (Figure 1a). In addition, a thick native passivation layer dominated by Li_2_CO_3_ is present both initially and after storage (Figure 1d,e). Because Li_2_CO_3_ possesses low ionic conductivity (10^−8^–10^−12^ S cm^−1^) and brittle mechanical characteristics [39, 40], it significantly increases interfacial impedance and accelerates interfacial degradation during cycling. Consequently, the poor surface quality and unstable interphase of extruded Li promote continuous SEI growth, severe void formation, and lithium dendrite propagation during cycling, ultimately leading to inferior electrochemical performance.

In contrast, thermally evaporated Li consists of high‐purity lithium with a smooth and dense surface morphology, which improves interfacial contact with LPSCl solid electrolyte and enables more homogeneous lithium plating/stripping. However, owing to the high chemical reactivity of freshly deposited lithium, gradual surface degradation still occurs during storage, leading to the formation of Li_2_CO_3_ species on the Li surface. During cycling, although the improved Li quality alleviates interfacial instability and suppresses dendrite growth to some extent, the unprotected PVD Li surface remains susceptible to continuous parasitic reactions and SEI growth. Furthermore, when the Li thickness is reduced, void formation and interfacial resistance become increasingly pronounced, resulting in deteriorating cycling stability.

To address these limitations, an optimized LiF passivation layer was deposited onto the evaporated Li (25 µm). In addition to the intrinsic advantages of thermally evaporated Li, the LiF layer effectively suppresses the formation of Li_2_CO_3_ during storage (Figure 1d), thereby improving long‐term surface chemical stability. During cycling, the LiF layer further mitigates parasitic side reactions between Li and the solid electrolyte (Figure 4), maintains stable interfacial contact, and provides high interfacial energy against lithium [41, 42, 43]. As a result, continuous SEI growth, void formation, and dendrite propagation are effectively suppressed (Figure 5), leading to significantly improved interfacial stability and long‐term electrochemical performance.

The selection of LiF as the interfacial layer is based on several key criteria relevant to stable Li metal anodes. First, LiF possesses excellent (electro‐)chemical stability against Li metal. Owing to its large negative Gibbs free energy of formation (−588 KJ mol^−1^) and high thermodynamic stability [21, 22], LiF is difficult to decompose, enabling the formation of a robust and persistent interphase, thereby contributing to the surface chemical stability of Li metal anodes. Second, LiF exhibits high mechanical robustness, with a reported shear modulus of ∼55.6 GPa and Young's modulus of ∼65 GPa [44], exceeding those of common SEI species such as Li_2_S (∼20–30 GPa), and LiCl (∼35–40 GPa) [45, 46]. Such a mechanically robust LiF interphase can better resist stress accumulation and dendrite penetration. Third, LiF possesses extremely low electronic conductivity (∼10^−10^ S cm^−1^) [47], which effectively blocks electron transport and mitigates the decomposition of LPSCl and continuous SEI growth. Fourth, LiF exhibits relatively high interfacial energy with Li (73.28 meV Å^−2^) as reported [48], significantly higher than those of Li_2_S (19.01 meV Å^−2^) and LiCl (37.55 meV Å^−2^), contributing to its superior dendrite‐suppressing ability.

Notably, the thickness of the LiF layer should also be carefully optimized. The layer must be sufficiently thick to ensure complete surface coverage and interfacial uniformity, while excessive thickness would increase interfacial resistance because of the intrinsically low ionic conductivity of LiF (∼10^−8^ S cm^−1^) [38, 39]. In this work, a 65 nm LiF layer provides an optimal balance between interfacial stability, dendrite suppression, and Li^+^ transport.

Based on these considerations, the universal design principle for artificial interphases on Li metal anodes is that the interfacial layer should simultaneously regulate chemical stability, mechanical stability, electron transport, and Li deposition behavior. An effective interphase should suppress parasitic reactions and dendrite growth while maintaining homogeneous Li^+^ flux and acceptable interfacial transport kinetics. In addition, the interphase thickness should be carefully optimized to balance surface protection and ion‐transport resistance. Therefore, rational interphase design requires the synergistic optimization of interfacial chemistry, mechanics, and transport properties.

With prolonged lithium plating and stripping with large areal capacity (> 2 mAh cm^−2^, equivalent lithium thickness of 9.7 µm), the ability of the LiF layer to effectively maintain interfacial stability between the thin evaporated Li (25 µm) and SE became limited after 200 cycles. This indicates that further strategies, such as introducing lithiophilic layers on the Cu current collector and exploring other family of barrier layers to better mitigate both the SEI and dendrites formation, are required to enhance long‐term cycling performance.

## 3 Conclusions

In conclusion, this study systematically investigated the surface morphology, chemical composition, interfacial stability, and cycling performance of commercially extruded lithium, thermally evaporated lithium, and LiF‐passivated thermally evaporated lithium. The thickness dependence of evaporated lithium highlights the importance of a sufficient lithium reservoir to suppress void formation and compensate for the SEI formation. By integrating optimized LiF layers that stabilize the Li/SE interface, superior cycling performance was achieved with 25 µm evaporated lithium, outperforming even twice‐as‐thick commercially extruded Li (50 µm). These findings underscore the promise of thermally evaporated lithium anodes and demonstrate the effectiveness of LiF passivation strategy, offering a viable pathway for next‐generation all‐solid‐state batteries with high energy density and robust performance under demanding operating conditions.

## 4 Experimental Section

### 4.1 Battery Materials, PVD Lithium Deposition and Lithium Surface Passivation

The solid electrolyte (SE) Li_6_PS_5_Cl (LPSCl) (3 µm particle size) was purchased from INCHEMS Co., Ltd. The LPSCl powder was ball milled before pressing into pellets following our previous procedures [23, 32]. The following condition was applied: 5 ZrO_2_ balls (10 mm in diameter) with 500 mg LPSCl powder in a FRITSCH Planetary Micro Mill at 140 rpm for 1 h.

The LiNi_0.8_Co_0.1_Mn_0.1_O_2_ (NCM811) cathodes active material was sourced from MSE, and the conductive carbon additive C65 was from Imerys. The coating layer of LiNbO_3_ was applied to pristine NCM811 powders to enhance the interfacial stability between NCM811 and LPSCl, followed by the procedures described in our previous study [33]. The NCM811 powder was suspended in anhydrous ethanol to achieve a concentration of 0.8 g mL^−1^. To prepare the sol‐gel, a 1 M lithium ethoxide (Acros Organics) and a 0.5 M niobium ethoxide (Alfa Aesar purity 99.999%) were added in 1:1 molar ratio, resulting in LiNbO_3_ content of 1 wt% relative to NCM811. A 33 wt% hydrogen peroxide solution was added into the sol at a volumetric ratio of 1:40 relative to ethanol. Then, the resulting suspension was sonicated for 30 min at 80 kHz with 80% power using an Elmasonic P 60H sonicator, and calcinated at 350°C for 3 h with an increasing rate of 5°C min^−1^ under a constant oxygen flow of 25 L h^−1^. Finally, the cathode composite, consisting of LiNbO_3_‐coated NCM811, LPSCl, and C65 in a mass ratio of 70:27:3, was prepared by hand‐mixing in a mortar. All materials synthesis, electrode preparation, cell assembly, and electrochemical cycling were conducted in an Argon‐filled glovebox at room temperature.

The commercially extruded 50 µm thick lithium metal films were obtained from China Energy Lithium Co., Ltd. The thermally evaporated Li with varying thicknesses was deposited using a MBRAUN vacuum‐based vapor deposition system. The lithium granules were heated inside a cylindrical stainless‐steel crucible (diameter of 2.3 cm and height of 4.7 cm) under vacuum (7.10^−7^ mbar) until vaporization, followed by condensation onto a copper foil (20 µm thick). The evaporation rate was monitored and controlled at 20 Å s^−1^ using a quartz crystal microbalance (QCM) sensor and the INFICON SQC‐310 Series. To passivate the surface of lithium metal anodes, an electron beam evaporation process was conducted to deposit a lithium fluoride (LiF) layer. The LiF powder was purchased from Sigma‐Aldrich and filled into the crucible made of graphite. The electron beam source operated at an acceleration voltage of 3 kV and a current of 35 mA, within a vacuum chamber maintained at 3.5 × 10^−6^ mbar. The lithium foils were transferred between the glovebox and the ultra‐high vacuum system for surface passivation and XPS measurements under a controlled atmosphere using a specific transfer chamber, preventing any surface modification from exposure to air or moisture. On the other hand, the XPS measurements performed on the LiF‐coated lithium are performed in‐situ inside the ultrahigh vacuum cluster with a base pressure of 10^−9^ mbar [49].

### 4.2 Cell Assembly and Electrochemical Measurements

Bulk‐type ASSBs were assembled into a custom‐made cell following the cell assembly details described in our previous works [23, 32]. For the lithium symmetric cells, 60 mg LPSCl was loaded into a 7 mm diameter die and compacted using a hydraulic press at 380 MPa for 1 min. Subsequently, the SE pellets were transferred within the die under 50 MPa into the vacuum chamber (∼5.10^−1^ mbar) connected to the glovebox for sintering at 80 °C. A lithium edge protection, made of an insulating polymer ring (high‐density polyethylene: HDPE) with an outer diameter of 7 mm and an inner diameter of 5 mm was pressed on both sides of SE pellets at 25 MPa. The lithium metal was punched into 5 mm discs and pressed onto SE pellets at 25 MPa for 1 min. For the NCM811|LPSCl|Li full cells, 4.2 mg cathode composite (∼15 mg (NCM811) cm^−2^) was loaded and pressed onto SE pellets at 510 MPa for 1 min. The anode side was assembled following the same procedure as the symmetric cells. Subsequently, the assembled cells were closed at 20 MPa, rested at open circuit voltage (OCV) for 2 h and cycled inside the glovebox.

Galvanostatic cycling measurements were conducted on Li|Li symmetric cells at different current densities and areal capacities. Full cells were cycled at various C‐rates between 2.7 and 4.3 V vs. Li^+^/Li. A theoretical capacity of 200 mAh g^−1^ was used to calculate the applied current density, resulting in 1C equivalent to 3 mA cm^−2^ on the lithium metal anode. Electrochemical impedance spectroscopy (EIS) was performed using a Biologic VSP‐300 potentiostat in the frequency range from 10 mHz to 7 MHz with 10 mV amplitude. The Distribution of Relaxation Times (DRT) analysis was performed using RelaxIS software, version 3.0.23.26 (rhd instruments) to transform the EIS data from the frequency domain into the time domain. A regularization parameter λ of 10^−5^ was applied to ensure sufficient peak separation resolution without overfitting. Additionally, a shape factor µ of 0.5 was used across all DRT pattern computations in this study.

### 4.3 Three‐Electrode Cell Assembly and Measurements

The NCM811|LPSCl|Li three‐electrode cell was assembled in a polyetheretherketone (PEEK) sleeve of 12 mm inner diameter using a uniaxial press inside the Ar‐filled glovebox. We employed a commercial cell (CompreCell 12 DP‐3e, rhd instruments GmbH) designed to allow the integration of a reference electrode [34]. The Gold‐plated tungsten wire (denoted Au/W wire) with 30 µm diameter and 3–5 wt.% gold coverage (Goodfellow Cambridge Ltd.) was used as the basis of the reference electrode (RE). The wire was cut into a 5 cm long segment and inserted into the PEEK sleeve (inner diameter 12 mm) through the two pinholes on the sleeve wall and fixed by an O‐ring (14 × 1 mm) from the outer wall of the sleeve. 180 mg of LPSCl was compressed evenly on both sides of the wire under a uniaxial pressure of 510 MPa, forming a 12 mm diameter LPSC pellet as a separator with a thickness of approximately 1 mm. The working electrode (WE) is composed of NCM811 cathode composite (12.3 mg) and compressed at 510 MPa on one side of the LPSCl pellet. A metallic lithium counter electrode (CE) with a 9 mm diameter (50 µm thick) obtained from China Energy Lithium Co., LTD was punched out and pressed onto another side of the LPSCl pellet at 25 MPa. A stack pressure of 20 MPa was applied during cycling.

The Au/W wire was in situ lithiated with the excess lithium source from the lithium metal counter electrode to form a thin lithium metal reference electrode with a stable potential. A lithiation current of 2.5 µA (current density 0.22 mA cm^−2^(RE)) was applied for 2 h, during which approximately 1 h of lithium metal plating at stable voltage plateau of −0.01 V was performed, corresponding to ∼1.1 µm lithium thickness.

The galvanostatic cycling and impedance measurements of the NCM811|LPSC|Li three‐electrode cell was conducted using a BioLogic VSP‐300 potentiostat. A constant current of 0.064 mA (current density 0.1 mA cm^−2^(CE)) was applied during the cycling with the upper and lower cut‐off potentials set as 4.3 V and 2.7 V, respectively. The impedance of the three‐electrode cell was measured between WE & CE, WE & RE, and CE & RE, after resting at OCP for 1 h after charge. The impedance was recorded from 10 mHz to 7 MHz with 10 mV amplitude.

### 4.4 X‐Ray Photoelectron Spectroscopy (XPS)

XPS measurements were conducted on a VG ESCALAB 220iXL spectrometer (Thermo Fisher Scientific) using focused monochromatized Al Kα radiation (1486.6 eV) with a beam size of ∼500 µm^2^ (power of 150 W). The spectrometer was pre‐calibrated by performing a measurement on a clean silver surface, whereby the Ag 3d_5/2_ peak was aligned to a binding energy of 368.25 eV with a full width at half‐maximum of 0.78 eV at a pass energy of 20 eV. Survey spectra were recorded with a dwell time of 50 ms, using a pass energy of 50 eV in steps of 0.5 eV. For high‐resolution spectra, these parameters were adjusted to 20 and 0.05 eV, respectively. Peak deconvolution was performed using CasaXPS software, applying the sum of Gaussian (70%) and Lorentzian (30%) line shapes after a Shirley‐type background subtraction. No charge compensation was applied, and the binding energy calibration was referenced to the C 1s core level located at 284.8 eV. The estimated atomic percentage of the elements is calculated using Avantage software from Thermo Fisher.

### 4.5 Scanning Electron Microscopy (SEM)

SEM measurements were conducted in a field emission gun equipped with SEM Zeiss ULTRA 55 using the Secondary Electron Emission Detector of 5 kV accelerating voltages and ∼5 mm working distance. Energy‐dispersive X‐ray spectroscopy (EDX) mapping was conducted at 5 kV using an Oxford Ultim Extreme detector, with data processing performed in AZtec software.

## Author Contributions

**Jinsong Zhang**: conceptualization, investigation, Writing – original draft, methodology, validation, visualization, data curation, Writing – review and editing. **Linfeng Xu**: methodology, data curation, writing – review and editing. **Robin N. Wullich**: methodology, data curation, writing – review and editing. **Thomas J. Schmidt**: writing – review and editing, supervision, validation. **Mario El Kazzi**: funding acquisition, conceptualization, visualization, writing – review and editing, project administration, supervision, resources, validation.

## Conflicts of Interest

The authors declare no conflicts of interest.

## Supporting information




**Supporting File**: advs76456‐sup‐0001‐SuppMat.docx.

## Data Availability

The data that support the findings of this study are openly available in Zenodo at https://doi.org/10.5281/zenodo.18338629, reference number 18338629.
