# Supplementary material for: Enhancing All‐Solid‐State Batteries Performance Through Thickness Control and Surface Passivation of Thermally Evaporated Lithium Metal Anodes
Source: Adv Sci (Weinh). 2026 Jul 13;13(54):e76456. doi: 10.1002/advs.76456 (PMC13360118; doi:10.1002/advs.76456)
Supplement: Supplementary file 1 [file ADVS-13-e76456-s001.docx]

**Supporting information**

**Enhancing All-Solid-State Batteries Performance through Thickness Control and Surface Passivation of Thermally Evaporated Lithium Metal Anodes**

Jinsong Zhang,^1^ Linfeng Xu,^1^ Robin N. Wullich,^1^ Thomas J. Schmidt,^1,2^ Mario El Kazzi^1,*^

**Affiliation(s):**

^1^ PSI Center for Energy and Environmental Sciences, Paul Scherrer Institute, 5232 Villigen, Switzerland

^2^ Institute for Molecular Physical Science, ETH Zurich, 8093 Zurich, Switzerland

Figure S1. Cross sectional SEM images performed on thermally evaporated lithium metal with thickness of 50 μm.

Figure S2. F 1s XPS spectra acquired on the LiF-coated evaporated lithium at pristine stage.

Figure S3. XPS survey spectra acquired on (a) commercially extruded Li, (b) thermally evaporated Li, (c) LiF-passivated evaporated Li, at the pristine stages and after storage for 7, 14, 28 days in glovebox.

Figure S4. Atomic percentages of C, O, Li, and F for different lithium films, (a) commercially extruded Li, (b) thermally evaporated Li, (c) LiF-passivated evaporated Li, at the pristine stages and after storage for 7, 14, 28 days in glovebox.

Figure S5. XPS O 1s spectra acquired on (a) commercially extruded Li, (b) thermally evaporated Li, (c) LiF-passivated evaporated Li, at the pristine stages and after storage for 7, 14, 28 days in glovebox.

Figure S6. XPS Li 1s spectra acquired on (a) commercially extruded Li, (b) thermally evaporated Li, (c) LiF-passivated evaporated Li, at the pristine stages and after storage for 7, 14, 28 days in glovebox.

Figure S7. XPS F 1s spectra acquired on LiF-passivated evaporated Li, at the pristine stages and after storage for 7, 14, 28 days in glovebox.

Figure S8. Fraction of lithium stripped from the total lithium reservoir for thermally evaporated lithium with various thicknesses, after complete depletion of electrochemically active lithium and after stripping 0.5 mAh cm^-2^ of lithium, corresponding to point C and point B in Figure 2.a, respectively.

Figure S9. DRT patterns corresponding to EIS spectra of Li|Li symmetric cells at point C in Figure 2.b. orange area indicates resistance of solid electrolyte (R_SE_), red area indicates resistance of void (R_Void_), and green area indicates resistance of solid electrolyte interphase (R_SEI_).

Figure S10. The charge-discharge voltage profiles of long-term cycling tests for the NCM811|LPSCl|Li full cells at 1 mA cm^-2^, corresponding to Figure 3.a. (a) Li_12.5 μm, (b) Li_25 μm, (c) Li_37.5 μm, and (d) Li_50 μm.

Figure S11. Comparison of thermally evaporated Li (25 μm thick) anodes coated with LiF layers of varying thicknesses: (a-c) EDX maps of F Kα signals showing the areal coverage of F on evaporated lithium surfaces, (d-f) GKD maps of the F Kα signals showing the LiF layer uniformity. For comparison, the density values were normalized by the median F Kα signal intensity on the Li metal anodes, which effectively removes the information on the absolute intensity but displays the inhomogeneity with a high sensitivity.

Figure S12. DRT patterns corresponding to EIS spectra of NCM811|LPSCl|Li full cells after 1^st^, 50^th^, 200^th^, 300^th^ cycle at 1 mA cm^-2^ in Figure 4.a, red area indicates resistance of void (R_Void_), blue area corresponds to the combined SEI and cathode resistance (R_SEI_ / R_Cathode_), featuring three distinct peaks (R_1_, R_2_, R_3_), and purple area indicates resistance of cathode (R_Cathode_).

Figure S13. DRT patterns measured on the first charge using three-electrode cell performed on the NCM811|LPSCl|Li full cell employing lithium metal as reference electrode. It includes impedance between NCM811 working electrode (WE) and reference electrode (RE), lithium metal counter electrode (CE) and reference electrode, and NCM811 working electrode (WE) and lithium metal counter electrode (CE).

Figure S14. DRT analysis showing the evolution of cathode resistance (R_Cathode_) at a time constant of 10^-1^ s with cycle number in NCM811|LPSCl|Li full cells.

Figure S15. Cycling performance of NCM811|LPSCl|Li full cell employing evaporated Li (25 μm thick) anodes with/without a 65 nm LiF passivation layer, at a current density of 1.5 mA cm^-2^.

Figure S16. The charge-discharge voltage profiles of long-term cycling tests for the NCM811|LPSCl|Li full cells at 3 mA cm^-2^, with thermally evaporated Li (25 μm thick) anodes coated with a 65 nm LiF passivation layer, corresponding to Figure 5.b.

Table_S1. Comparative table summarizing recent studies using Li_6_PS_5_Cl-based solid electrolytes, lithium metal anodes, and NCM-based cathodes, highlighting the cycling performance of full cells.

| Electrolyte | Anode | Cathode | Current density mA/cm^2^ | Areal capacity mAh/cm^2^ | Cycle life | Capacity retention % | Cycle  condition | Ref. |
| --- | --- | --- | --- | --- | --- | --- | --- | --- |
| Li_6_PS_5_Cl  (Sb) | 30 - 50 μm  Li foil | LiNi_0.5_Mn_0.3_Co_0.2_O_2_  (LiNbO_3_ coating) | 0.72 | 1.25 | 450 | 84 | 1.7 MPa  75ºC | [1] |
| Li_6_PS_5_Cl | 60 μm  Li foil  (Li_2_Te) | LiNi_0.8_Co_0.1_Mn_0.1_O_2_ | 0.7 | 1.03 | 400 | 95.4 | 13 MPa  RT | [2] |
| Li_6_PS_5_Cl  (Li_3_TCA) | 50 μm  Li foil | LiNi_0.8_Co_0.1_Mn_0.1_O_2_ (LiNbO_3_ coating) | 0.9 | 1.65 | 500 | 47 | 20 MPa  RT | [3] |
| Li_6_PS_5_Cl | 30 μm  Li foil  (MgF_2_) | LiNi_0.7_Co_0.15_Mn_0.15_O_2_ | 0.78 | 1.7 | 800 | 82 | 15 MPa  30 ºC | [4] |
| Li_6_PS_5_Cl  (Sintered @ 80°C) | 50 μm  Li foil  (LiF) | LiNi_0.8_Co_0.1_Mn_0.1_O_2_ | 1  1.5 | 1.46  1.36 | 1500  450 | 75  91 | 20 MPa  RT | [5] |
| Li_6_PS_5_Cl  (Sintered @ 80°C) | 25 μm  Evaporated  Li foil  (LiF) | LiNi_0.8_Co_0.1_Mn_0.1_O_2_  (LiNbO_3_ coating) | 1 | 2.08 | 200  500 | 96  52 | 20 MPa  RT | This work |
| Li_6_PS_5_Cl  (Sintered @ 80°C) | 25 μm  Evaporated  Li foil  (LiF) | LiNi_0.8_Co_0.1_Mn_0.1_O_2_  (LiNbO_3_ coating) | 3 | 1.3 | 300 | 91 | 20 MPa  RT | This work |

**References**

1.	Šivavec, J., M. Klimpel, J.F. Baumgärtner, H. Zhang, B. Erdivan, R. Widmer, M.V. Kovalenko, and K.V. Kravchyk, *Low-pressure cycling of lithium metal anodes with argyrodite solid-state electrolytes enabled by an Sb-based interfacial layer.* Energy Storage Materials, 2026. **84**: p. 104760.

2.	Hao, H., Y. Liu, S.M. Greene, G. Yang, K.G. Naik, B.S. Vishnugopi, Y. Wang, H. Celio, A. Dolocan, W.-Y. Tsai, R. Fang, J. Watt, P.P. Mukherjee, D.J. Siegel, and D. Mitlin, *Tuned Reactivity at the Lithium Metal–Argyrodite Solid State Electrolyte Interphase.* Advanced Energy Materials, 2023. **13**(46): p. 2301338.

3.	Braks, L., J. Zhang, A. Forster, P. Fritz, J. Oh, M. El Kazzi, J.W. Choi, and A. Coskun, *Interfacial Stabilization by Prelithiated Trithiocyanuric Acid as an Organic Additive in Sulfide-Based All-Solid-State Lithium Metal Batteries.* Angewandte Chemie International Edition, 2024. **63**(35): p. e202408238.

4.	Lim, H., S. Jun, Y.B. Song, K.H. Baeck, H. Bae, G. Lee, J. Kim, and Y.S. Jung, *Rationally Designed Conversion-Type Lithium Metal Protective Layer for All-Solid-State Lithium Metal Batteries.* Advanced Energy Materials, 2024. **14**(12): p. 2303762.

5.	Zhang, J., R.N. Wullich, T.J. Schmidt, and M. El Kazzi, *Synergistic Effects of Solid Electrolyte Mild Sintering and Lithium Surface Passivation for Enhanced Lithium Metal Cycling in All-Solid-State Batteries.* Advanced Science, 2026. **n/a**(n/a): p. e21791.
